# Deep brain stimulation reduces (nocturnal) dyskinetic exacerbations in patients with ADCY5 mutation: a case series

**DOI:** 10.1007/s00415-020-09871-8

**Published:** 2020-07-09

**Authors:** Ana Luísa de Almeida Marcelino, Tina Mainka, Patricia Krause, Werner Poewe, Christos Ganos, Andrea A. Kühn

**Affiliations:** 1 Movement Disorder and Neuromodulation Unit, Department of Neurology, Charité Campus Mitte, Charité, University Medicine Berlin, Chariteplatz 1, 10117 Berlin, Germany; 2 Berlin Institute of Health (BIH), 10178 Berlin, Germany; 3 Department of Neurology, Medical University Innsbruck, 6020 Innsbruck, Austria; 4 Berlin School of Mind and Brain, Charité-University Medicine Berlin, 10117 Berlin, Germany; 5 NeuroCure Clinical Research Centre, Charité-University Medicine Berlin, 10117 Berlin, Germany; 6 DZNE, German Centre for Degenerative Diseases, 10117 Berlin, Germany

**Keywords:** ADCY5, Hyperkinetic movement disorder, Dyskinesia, Deep brain stimulation, Globus pallidus internus

## Abstract

Mutations in the ADCY5 gene can cause a complex hyperkinetic movement disorder. Episodic exacerbations of dyskinesia are a particularly disturbing symptom as they occur predominantly during night and interrupt sleep. We present the clinical short- and long-term effects of pallidal deep brain stimulation (DBS) in three patients with a confirmed pathogenic ADCY5 mutation. Patients were implanted with bilateral pallidal DBS at the age of 34, 20 and 13 years. Medical records were reviewed for clinical history. Pre- and postoperative video files were assessed using the “Abnormal Involuntary Movement Scale” (AIMS) as well as the motor part of the “Burke Fahn Marsden Dystonia Rating Scale” (BFMDRS). All patients reported subjective general improvement ranging from 40 to 60%, especially the reduction of nocturnal episodic dyskinesias (80–90%). Objective scales revealed only a mild decrease of involuntary movements in all and reduced dystonia in one patient. DBS-induced effects were sustained up to 13 years after implantation. We demonstrate that treatment with pallidal DBS was effective in reducing nocturnal dyskinetic exacerbations in patients with ADCY5-related movement disorder, which was sustained over the long term.

**Electronic supplementary material:**

The online version of this article (10.1007/s00415-020-09871-8) contains supplementary material, which is available to authorized users.

Supplementary material: © The authors.

The supplementary material in this article is not included in the Creative Commons license and is protected by copyright. For permissions please contact the authors.

## Introduction

The phenotypic presentation of patients carrying pathogenic ADCY5 gene mutations includes a wide range of movement disorders, most notably chorea, dystonia and myoclonus [1–3]. Two further core features are axial hypotonia and episodic exacerbations of hyperkinesias often referred to as “ballistic bouts” or “spells”, which predominantly occur during drowsiness or sleep [1, 3, 4]. Even though these episodic exacerbations are often clinically misdiagnosed as nocturnal frontal lobe or other types of focal epilepsy, ictal EEG abnormalities are absent in most cases. Further features may include hypersalivation, spasticity and oculomotor abnormalities. Cognitive and intellectual function are mostly preserved. The disease was first described in 1978 as “familial essential (benign) chorea” [5] and renamed “autosomal dominant familial dyskinesia and facial myokymia” in 2001 in a description of the same family over five generations [6]. Since the discovery of the mutated gene responsible for this phenotype in 2012 by whole exome sequencing within this same family [7], more than 70 cases have been described until today. Medical treatment remains largely unsatisfactory. A mild decrease of dyskinesia with acetazolamide [6, 8], clonazepam and clobazam [1, 3] has only been reported in individual cases.

The benefit of deep brain stimulation (DBS) as a treatment for isolated generalized and segmental dystonia is well established [9, 10] and there is also increasing evidence that choreatic movements in Huntington’s disease can improve after pallidal DBS [11, 12]. Until now, four cases of DBS as a treatment for ADCY5-related movement disorder have been described [13, 14]. In one case series, patients received pallidal stimulation at 3, 8 and 32 years of age and experienced mild improvement [13]. Long-term follow-up was available for a maximum of 3 years. Thus, evidence regarding the overall efficacy for DBS in ADCY5-associated movement disorders is scarce. More specifically, it is not clear which symptoms of this complex disorder respond best to DBS and how long effects sustain. Here, we report on three patients who have received pallidal DBS surgery for ADCY5-related disorder and evaluate the DBS effect on a general and symptom-specific level at short- and long-term follow-up.

## Methods

Three patients with genetically confirmed ADCY5-related hyperkinetic movement disorder treated with DBS of the Globus pallidus pars interna (GPi) at Charité—Universitätsmedizin Berlin were identified. All available documents of the respective patients were reviewed for clinical history, examination and response to treatment to account for a most detailed report of the events (clinical details are summarized in Table 1). All patients underwent clinical examination with video assessment for long-term follow-up (Case 1: 3 months, 3, 6, 9 and 13 years after surgery; Case 2: 6 months, 3 and 8 years; Case 3: 3 months, 1 and 3 years postoperatively). Pre- and postoperative video files were reviewed by a movement disorders experienced neurologist (ALAM) and the “Abnormal Involuntary Movement Scale” (AIMS) as well as the motor part of the “Burke Fahn Marsden Dystonia Rating Scale” (BFMDRS) were applied. For the AIMS, no points were given for the section “Tongue” (in I Facial and Oral Movements), as tongue movements could not be assessed in all videos. Exemplary video clips are provided as online resources to this manuscript (Videos 1–3 for cases 1–3, respectively). Patients (and for patient two her legal guardian) provided written informed consent for publication.

**Table 1:** Demographic and clinical characteristics of included patients

	**Case 1 (m)**	**Case 2 (m)**	**Case 3 (f)**
Age at report/at surgery (years)	47/34	28/20	16/13
Mutation details	c1252C > T; p.Arg418Trp		
Age at onset	6 months	6 months	3–4 months
Predominant motor feature	Choreoathetosis > Dystonia	Dystonia > Myoclonus > Choreoathetosis	Myoclonus > Dystonia > Choreoathetosis
Triggers for dyskinetic exacerbations	Emotional stress, drowsiness/sleep	Drowsiness/sleep, quick change of muscle tone	Emotion/stress, movement, temperature changes, rarely at night
Medication tried before DBS	Carnitine, dopamine, carbamazepine, baclofen, tetrabenazine, trihexyphenidyl, diazepam, acetazolamide, levetiracetam	Carbamazepine, levetiracetam, gabapentine, valproate, acetazolamide, topiramate, clonazepam, diazepam, pramipexol, levodopa, botulinum toxin	Levodopa, carnitine, carbamazepine, clonazepam
Subjective improvement through DBS/improvement of nocturnal exacerbation	GCI 60%/ 80% improvement of nocturnal exacerbations	GCI 50%/ 90% improvement of nocturnal exacerbations	GCI 40–50%/ n.a
AIMS Score at BL and last FU	29/21 (13YFU)	20/18 (8YFU)	22/20 (3YFU)
BFMDRS at BL and last FU	67/58 (13YFU)	56/56 (8YFU)	49/47.5 (3YFU)
Current stimulation parameters	Left GPi: contacts 10-, 11-, case + ; 1.9 V; right GPi: contacts 2-, 3- case + , 1.5 V; 90 μs, 170 Hz	Left GPi: contact 9-, case + , 2.6 V; right GPi: contact 1-, case + , 2.5 V; 90 μs, 180 Hz	Left GPi: contact 4-, case + , 1.5 mA; right GPi contact 1-, 2-, case + , 2.2 mA; 90 µs, 130 Hz

*AIMS* abnormal involuntary movements scale, *BFMDRS* burke fahn marsden dystonia rating scale, *BL*  baseline, *FU*  follow-up, *GCI*  global clinical improvement, *GPi*  Globus pallidus pars interna, *m*  male, *f*  female, *n.a.* not applicable

## Results

### Case 1

Patient 1 is a 47-year-old man who had been treated at the Charité-Universitätsmedizin Berlin since infancy. After an uneventful birth and early development, abnormal movements were first noted at the age of 6 months and subsequent motor development was delayed with poor axial and head control. Generalized choreoathetosis was present at rest and increased with voluntary movement and emotional stress. The movement disorder was marked by short exacerbations (< 1 min) during the day. Fine and gross motor skills were impaired, he never learned to write and required assistance in feeding, dressing and daily hygiene. The movement disorder remained constant during childhood. He had a normal active vocabulary, but speaking required effort and was dysarthric. There were no signs of cognitive impairment, after school he worked in electronic data processing. At 18 years of age he started having motor attacks with increased muscle tone, flexion of the arms and extension of the legs lasting a few minutes up to 1 h. Attacks would appear mainly during drowsiness or at night, interrupting sleep over five times per night. Family history was incomplete as both parents died during his childhood (alcohol abuse), but negative for neurologic disorders.

Extensive work-ups throughout the years including cranial MRI and computed tomography, cerebrospinal fluid (CSF) analysis, metabolic screening, muscle biopsy, nerve conduction studies, electromyography, and ophthalmologic examinations were inconclusive. Even though repetitive electroencephalographic recordings (including sleep-deprival and continuous recordings over 24 h) failed to reveal epileptic discharges, sleep-related hypermotor epilepsy was suspected. Several medications were introduced to treat the movement disorder and the nocturnal dyskinetic episodes, but with unsatisfactory effect (see Table 1 for detailed list).

Deep brain stimulation surgery was performed at the age of 34. Clinical examination prior to implantation revealed severe permanent generalized choreoathetosis more pronounced on the upper extremities and the right side. The trunk and the feet were fixated to the wheel-chair with belts. Facial dyskinesia was more evident in the lower part of the face with permanent choreatic movements of tongue and perioral muscles. There was no dysphagia, speech was effortful and dysarthric. Cervical dystonia was intermittent, mainly induced by action and predominantly phasic. Action-induced dystonia was also present in the limbs (upper > lower). Voluntary control of specific movements was impaired, for instance he was not able to close his eyes tightly (maximum short blinking) or hold both arms stretched out at the same time. Limb muscle strength, tone and deep tendon reflexes were normal. Axial hypotonia was evident, standing and ambulation were not possible. Medtronic 3389–28 electrodes were placed bilaterally in the GPi and a Medtronic Kinetra pulse generator (implantable pulse generator, IPG) was implanted. The preoperative AIMS and motor part of the BFMDRS as assessed retrospectively per video comprised 29 (out of 36 points, as tongue movements were not assessable in all videos) and 67 (120) points, respectively. After surgery, the patient reported on an improvement of involuntary hyperkinetic movements, fine motor skills of the hands and speech. This was reflected in a sustained reduction of the AIMS and BFMDRS (see Table 1 for details). Subjective rating was reported at 2 and 13 years after implantation with a subjective general improvement of 60%, reduction of the exacerbations at night up to 80% and 50% reduction of the hyperkinetic movements during the day.

Seven years after implantation, a technical defect of the stimulator led to progressive worsening of daytime choreoathetosis and increased frequency of nocturnal attacks. A similar aggravation occurred 5 years later after removal of the IPG over 4 weeks due to infection. In both cases, replacement/re-implantation and activation of the stimulation led to a full recovery. Genetic investigation at the age of 47 revealed a known pathogenic ADCY5 mutation and the diagnosis of ADCY-related disorder was confirmed.

### Case 2

Patient 2 is a 28 year-old-male who first presented to our outpatient clinic at the age of 16 years. He was born on term as the second of dizygotic twins through planned caesarean section. Pregnancy was normal and there were no reports of perinatal complications. At about 6 months of life a delay in motor development compared to his twin brother and axial hypotonia with poor head control were noticed as well as leg spasticity. Speech development was also delayed. At 8 months, he would sit in a “W” position. This sitting position was maintained for several years. At 2 years of age he would stand up independently. Ambulation was achieved in a crouched position with marked difficulty. Milestones such as unsupported sitting, standing with normal posture or walking were never reached. At the age of 2, he began having sudden attacks with flexion of the arms, extension of the legs and reclination of the head and/or the trunk. These attacks would occur up to 30 times a day, mainly during drowsiness or sleep, and would interrupt sleep. They were triggered by quick changes of muscle tone (active or passive) and had a duration of less than 1 min. There were no prodromal signs, no loss of consciousness, enuresis or encopresis. Family history was negative for neurological conditions, especially regarding the dizygotic twin brother as well as a 2 year older brother.

Prior to referral to our outpatient clinic, a broad clinical and genetic work-up had been performed: cerebral MRI, extensive metabolic screening and repetitive EEGs had shown normal results. Sensory- and motor-evoked potentials of the lower limbs were indicative of a central defect. Genetic testing was negative for spinocerebellar ataxias, generalized dystonia and Pantothenate kinase-associated neurodegeneration. The nocturnal attacks described above were misdiagnosed as sleep-related hypermotor epilepsy and treatment was initiated with antiepileptic medication. A detailed list is summarized in Table 1.

Clinical exam at the first visit showed a 16-year-old young man sitting in a wheel-chair with head support. He had narrow eyes, a long thin face and oromandibular dystonia (involuntary jaw opening) as well as perioral myoclonic jerks and tongue dyskinesia. Execution of voluntary facial movements was impaired (e.g. unable to close his eyes when asked or show a sad face). Speech was effortful and dysarthric. Cervical dystonia was intermittent and predominantly phasic. He had spastic tetraparesis more pronounced on the right side with normal deep tendon reflexes but positive pyramidal signs on both feet. All limbs were preferably held in a flexed position, dystonia was induced by action. Myoclonus was present at rest and also triggered by voluntary movement. Ambulation was possible for a few steps in a crouched position. Fine motor skills were significantly impaired. He had to be fed, needed help to wash himself and to dress. A systematic neuropsychological exam was normal. He completed secondary school and was trained to work in counselling for social inclusion.

Before DBS surgery, the patient took 4.5 mg clonazepam and 25 mg diphenhydramin at night. At the age of 20 years, he underwent neurosurgery with implantation of bilateral pallidal electrodes (Medtronic 3389–28) and a Medtronic Activa PC IPG. After the implantation, he reported on reduction of the nocturnal hyperkinetic episodes by 90%. The benzodiazepines were slowly reduced to 0.5 mg/night over the following months. He reported a general subjective improvement of the hyperkinetic disorder of about 50%. Retrospective assessment of videos revealed only a slight reduction in AIMS and no effect on BFMDRS (see Table 1 for details). Almost complete cessation of nocturnal hyperkinetic attacks persisted over the entire follow-up period. In addition to improved sleep, no specific improvements in activities of daily living were reported after DBS. There were six episodes with severe worsening of dystonic features and/or nocturnal attacks that led to admission through the emergency service of our hospital. Three times (3 months, 6 years and 6 and a half years after surgery), a technical defect was detected and either both connecting cables or connecting cables and leads were replaced. Three times (4, 7 and 8 years after surgery), worsening of the movement disorder was due to battery exemption and improved after replacement. The pathological ADCY5 mutation was first detected 8 years after DBS implantation.

### Case 3

Patient 3 was a 16 year old girl that first presented in our outpatient clinic at 12 years of age. She had been born preterm by emergency caesarean section due to pre-eclampsia. Respiratory support through CPAP (“continuous positive airway pressure”) was needed for 4 days as well as a nasogastric tube. After discharge, new-born development was normal until the fourth month of life, when axial hypotonia was first noticed. At 12 months, discrete dyskinetic movements began. Developmental milestones were only met partially and with delay: at the age of 2 years, she was able to crawl and at 2.5 years, she was able to walk unassisted yet in a clumsy way and with need for a wheel-chair for longer distances. With 2 years, first hyperkinetic exacerbations appeared, lasting up to 1 min and occurring several times per hour. These hyperkinetic attacks started suddenly, were triggered by strong emotions, movement, temperature changes (for instance during bathing) and occurred rarely at night. During childhood, these sudden movement spells increased in frequency. Other clinical features such as hypersalivation, dysarthria and oral/tongue dyskinesia were reported. There were no signs of cognitive impairment. Family history was negative for movement disorders. Extensive clinical work-ups prior to first visit at our outpatient clinic including cerebral MRI at 1 and 5 years of age, analysis of cerebral spinal fluid including neurotransmitter metabolism and screening for metabolic disorders had remained elusive. Genetic analyses for dystonia, paroxysmal dyskinesia or episodic ataxia were negative but revealed a compound heterozygote ATM mutation of unknown significance that was first considered responsible for the disorder. Re-evaluation at the age of 12 revealed the pathogenic ADCY5 mutation. Treatment with levodopa was initiated at age of two (13 mg/kg/day) and led to an increase of dyskinesia. Carnitine, carbamazepine and clonazepam had no effect on the movement disorder.

Clinical examination at first visit showed a slim, pre-pubertal 12-year-old girl with permanent generalized choreoathetosis that was superimposed by intermittent myoclonus and dystonia. Axial hypotonia with dropped head was evident mainly when trying to stand up and during ambulation. She could stand unsupported for a few seconds. She would walk > 10 m without aid in a clumsy and dystonic manner. Perioral and tongue dyskinesia were nearly permanent and comprised slow choreatic movements as well as short jerks. Speech was effortful and dysarthric. Even though there was no apparent facial palsy, voluntary control of facial mimics and tongue movement were impaired. Deep tendon reflexes were brisk, pyramidal signs were negative. Limb muscle tone was normal, muscle strength fulfilled 5/5 MRC (“Medical Research Council” Scale). During the exam, the movement disorder exacerbated for episodes of < 1 min duration without an obvious trigger. The patient was not taking any medication at that time.

At the age of 13, DBS electrodes were implanted bilaterally in the GPi and a rechargeable Boston Scientific Vercise RC impulse generator subcutaneously in the left upper thorax. Immediately after surgery a reduction of dyskinesia was observed and sustained for about 6–8 weeks. 3 months after surgery, monopolar review was performed and new parameters were set, leading to a decrease in the hyperkinetic baseline movement disorder as well as the episodic storms. More than that, the ability to sit freely and to walk as well as the involuntary tongue movements and voluntary tongue control were improved. There was no effect on hypersalivation or dysarthria. 2 years after implantation the patient reported improved gait as biggest achievement of the stimulation. Furthermore, sitting and standing as well as tongue and facial dyskinesia were reported with clinically meaningful improvement. The sudden attacks during the day had become rarer. At the last follow-up visit, 3 years after implantation, the baseline movement disorder had deteriorated slightly, which resolved after subtle changes in current settings.

Retrospective video ratings of the AIMS revealed a mild improvement of involuntary movements, no evident improvement was assessed by the BFMDRS (see Table 1 for details). The girl and her father reported a subjective general improvement of 40–50% after DBS. 1 year after surgery, the battery of the IPG emptied completely due to technical problems. With delay of about 1 week involuntary movements increased leading to a visit of the emergency department. The transient worsening of the movement disorder resolved after proper charging of the IPG.

## Discussion

Here, we present long-term follow-up data of three patients with genetically confirmed ADCY5-related disorder who underwent pallidal deep brain stimulation at 34, 20 and 13 years of age due to severe hyperkinetic movements with episodic exacerbations. All cases showed mixed movement disorder phenotypes comprising chorea, dystonia and myoclonus, in line with previous descriptions of this genetic entity [1, 3, 5, 6]. Core features such as axial hypotonia, facial dyskinesia and episodic exacerbations of the generalized movement disorder were present in all patients [2]. The latter was very disabling for all patients, particularly in cases 1 and 2, in whom episodes occurred mainly during drowsiness or at night, hindered falling asleep or led to several interruptions of sleep during the night. Pallidal DBS led to a general subjective improvement of 40–60% with almost complete cessation of nocturnal hyperkinetic episodes. Retrospective video rating of the AIMS and BFMDRS revealed a mild reduction of involuntary movements during DBS in all patients that was sustained over the years and reduction of dystonia in one patient. The two patients who suffered from nocturnal dyskinetic exacerbations reported a specific improvement of 80 and 90% at night (cases 1–2, respectively). In case 2, this improvement was associated with postoperative reduction of clonazepam from 4.5 to 0.5 mg daily. Reported individual improvements of hyperkinetic movements, fine motor skills (case 1) or standing and walking (case 2) contributed to patient satisfaction after DBS, but all patients remained largely dependent in their activities of daily living. Over the years following stimulation, defect of stimulator or battery exemption were associated with sudden worsening of the baseline movement disorder and dyskinetic exacerbations in all three patients, which could be reversed after reestablishing normal functioning of stimulation.

In the present study, improvement of hyperkinetic episodic exacerbations was one of the main contributors to patient satisfaction after pallidal DBS, which was especially evident in the cases where they appeared at night and disrupted sleep. In the first published case series, Dy and colleagues described a decrease in amplitude and frequency of dyskinetic episodes in all three patients and “much improved sleep maintenance” in only one patient [13]. The other patient described in the literature also had “significant improvement of the dystonic episodes” and sleep disorder was described as “not persistent” but it remains unclear if there was a temporal association to DBS implantation [14]. As discussed by Dy et al., decrease of amplitude and frequency of baseline movement disorder and episodic exacerbations might facilitate falling asleep and reduce sleep interruptions [13, 15]. Another possibility is that sleep disorder is not (solely) a consequence of the movement disorder, but an independent symptom in ADCY5-related disorders. The basal ganglia are known to be involved in the regulation of sleep and wakefulness [16]. For instance, evidence suggests that activation of adenosine receptors in the Ncl. accumbens promotes sleep, whereas stimulation of dopamine receptors mediates behavioural arousal [16, 17]. The specific expression of ADCY5 in the Ncl. accumbens and its role as a second messenger of intracellular processes may implicate a sleep disturbance as consequence of a gain of function mutation in ADCY5-associated movement disorders [18]. Interestingly, caffeine, an antagonist of adenosine receptors in the Ncl. accumbens, has been shown to improve sleep disorder in individual cases of ADCY5-related disorders [19, 20]. Furthermore, lesioning the globus pallidus externa (GPe) in rats causes insomnia [21], whereas DBS of the same nucleus in rats [22] and in humans [23] promotes sleep. Even though several studies have found improved sleep quality, efficiency and duration in Parkinson’s disease patients undergoing DBS, it remains unclear if this is due to improved motor symptoms or a direct effect on sleep/wakefulness centres [24]. An indirect effect on GPe output has been hypothesized [22].

Our report provides a thorough analysis of symptom-specific effects with long-term results of DBS in ADCY-related disorder. Although all patients were satisfied with the general effect of pallidal DBS, motor scales failed to reflect this improvement: first, the scales used were not developed for this condition (AIMS for tardive dyskinesia and BFMDRS for generalized dystonia) and only address partial aspects of this complex phenotype. Second, all our patients scored rather high in both scores and a partial reduction of symptoms would not necessarily lead to a significant reduction in the overall score. Of note, in our patients, the clinically meaningful reduction of hyperkinetic episodes contributed essentially to the overall subjective improvement of about 40–60%, yet frequency and intensity of hyperkinetic (nocturnal) episodes are not considered in any of these scores. Even though ADCY5-related disorders are monogenic, patients present with phenotypic pleiotropy and distribution of predominant features may vary considerably [1, 3], as we observe in this case series. Furthermore, clinical response to DBS in other hyperkinetic disorders such as dystonia often vary in time with phasic/mobile dystonia showing a prompter response and according to the affected body area [25–27]. Thus, phenotypic pleiotropy might contribute to response variability following treatment with pallidal DBS in our patients [28]. Clinical standards for assessing complex movement disorders will be of major importance and larger cohorts are needed to evaluate which patients will benefit from pallidal DBS and finally to understand the symptom-specific network alteration.

Gathering more evidence for symptom-specific motor improvement with DBS in patients with ADCY mutations is of high relevance since pathogenic ADCY5 mutations might account for over 10% of early-onset non-progressive hyperkinetic movement disorders featuring chorea alone or combined with dystonia and/or myoclonus [8]. As it has recently been discovered, there might be a high number of undiagnosed cases. Medical treatment is widely ineffective and DBS has only been performed in a very limited number of patients. Increasing knowledge on treatment efficacy including long-term results will contribute to more detailed informed consent and improve patient selection as well as increased understanding of DBS as a treatment option for complex movement disorders.

## Electronic supplementary material

Below is the link to the electronic supplementary material. Supplementary material: © The authors. The supplementary material in this article is not included in the Creative Commons license and is protected by copyright. For permissions please contact the authors.

Video 1: This video shows patient one preoperatively, three months, three, nine and thirteen years postoperatively. Preoperatively, choreoathetosis predominates on the upper limbs at rest and is more pronounced on the right side. Action-induced dystonia and facial dyskinesia (mainly choreatic) are also present. After surgery there is an evident reduction of abnormal involuntary movements that is sustained up to the last follow-up, 13 years after surgery. Interestingly, after surgery the left body side is more affected, in contrast to prior to DBS. (MP4 109677 kb).

Video 2: Video recordings of patient two preoperatively, six months, three and eight years after pallidal DBS. Dystonia and myoclonus are the most prominent features. Perioral dyskinesia predominantly as short jerks, spasticity of the legs and axial hypotonia are also present. (MP4 109133 kb).

Video 3: This video depicts patient three preoperatively as well as twelve months and three years after bilateral pallidal DBS. On the first segment at twelve years of age the choreatic movements exacerbate for about 1min, corresponding to a typical exacerbation that occurred up to 10 times a day. The patient reported gait as most improved feature after stimulation. (MP4 451804 kb).
